# An Intelligent Platform for Software Component Mining and Retrieval

**DOI:** 10.3390/s23010525

**Published:** 2023-01-03

**Authors:** Nazia Bibi, Tauseef Rana, Ayesha Maqbool, Farkhanda Afzal, Ali Akgül, Manuel De la Sen

**Affiliations:** 1 Department of Computer Software Engineering, National University of Sciences and Technology, Islamabad 44000, Pakistan; 2 Department of Humanities and Basic Sciences, National Universityof Sciences and Technology, Islamabad 44000, Pakistan; 3 Departmentof Computer Science and Mathematics, Lebanese American University, Beirut 1102 2801, Lebanon; 4 Department of Mathematics, Art and Science Faculty, Siirt University, Siirt 56100, Turkey; 5 Department of Mathematics, Mathematics Research Center, Near East University, Near East Boulevard, Mersin 10, Nicosia 99138, Turkey; 6 Department of Electricity and Electronics, Institute of Research and Development of Processes, Faculty of Science and Technology, University of the Basque Country, 48940 Leioa, Bizkaia, Spain

**Keywords:** code reuse, recommendation systems, code recommendation, component-based software development, intelligent decision support system

## Abstract

The development of robotic applications necessitates the availability of useful, adaptable, and accessible programming frameworks. Robotic, IoT, and sensor-based systems open up new possibilities for the development of innovative applications, taking advantage of existing and new technologies. Despite much progress, the development of these applications remains a complex, time-consuming, and demanding activity. Development of these applications requires wide utilization of software components. In this paper, we propose a platform that efficiently searches and recommends code components for reuse. To locate and rank the source code snippets, our approach uses a machine learning approach to train the schema. Our platform uses trained schema to rank code snippets in the top k results. This platform facilitates the process of reuse by recommending suitable components for a given query. The platform provides a user-friendly interface where developers can enter queries (specifications) for code search. The evaluation shows that our platform effectively ranks the source code snippets and outperforms existing baselines. A survey is also conducted to affirm the viability of the proposed methodology.

## 1. Introduction

Robots are programmable machines that are now frequently seen and used in many areas. Therefore, these machines require quick development because they are typically employed to perform tasks quickly, effectively, and repetitively. The goal of robotic research has traditionally been to develop tools that can help people perform their tasks more efficiently. This objective has not changed much over time. However, coding robot-oriented systems is a very time-consuming and tiresome process if done from scratch. Because of this, programmers look for shortcuts and tend to reuse the code [1]. Most of the time, the programming of well-defined problems is carried out via a simple look-up [2]. The programmer scans the local code repository, follows it by a search in other repositories, and then diligently copies and pastes the found code. Due to the large collection of codes available for use, it is more likely that the users will find what they need for their particular task [3,4]. The practice of the code reuse has become popular after the introduction of software forges, such as Google Code and GitHub. StackOverflow and other “question and answer” (Q & A) sites also made code reuse easier and more widespread. Now users can readily look up the code they require, find useful ideas, and often put some existing code to use. The open-source ideology offers more to the users than just reusing existing code, it allows them to put up their high-quality code that would be available for others to reuse in web applications, such as Pastebin [5], GitHub Gist [6], and Codeshare [7].

Large-scale code reuse is gaining popularity because of its potential to save the programmer’s time and effort. Source code that is reused extensively can serve as a guide to developers because this code has already been implemented and requires less effort to maintain [8]. The degree to which the source code is used can also help to rank the functionality of relevant candidates [9]. Moreover, extensively reused codes and projects have distinguishing characteristics that set them apart from low-reuse projects. Some of these attributes may guide new projects that are striving to develop code that has considerable reuse potential.

In software development, the imperative task is to provide cost-efficient and high-quality software at a given time. An important approach to accomplish this goal is “Code Reuse”, which has been accepted by the majority of software development organizations [10]. The reused code can be obtained from many different sources such as source code of open-source software, third-party libraries, and Q&A websites, such as StackOverflow [11,12]. In recent years, application development has become very popular [13] and requires code reuse. However, the major problem with code reuse is that programmers often use the available code snippets in an ad hoc manner during application development.

A lot of work has been done on the topics of mobile development (e.g., [14,15], code reuse (e.g., [16,17]), and repositories (e.g., [3,18,19]). Repositories contain source code components with high-end functionality available for the developers’ use. Instead of building a new application feature developers tend to reuse the existing functionality of applications [20] that are already available in repositories. There are a lot of software component repositories, such as SourceForge and GitHub that contain software components for the diversified needs of app developers, software engineers, and usability experts [21]. Data in repositories are increasing exponentially and finding the relevant component to implement the application’s logic is challenging and time-consuming [22]. There is a need to make the process simple for developers to locate and identify a certain component. Our proposed platform addresses this issue by facilitating developers in locating desired components.

### 1.1. Role of Proposed Framework in the Development of Robotics and Internet of Things (IoT)-Based Systems

The proposed platform also facilitates the development of robotics, sensors, and IoT-based systems. The basic difference between IoT and robotic devices is that IoT devices are programmed to carry out specific tasks whereas robotic devices are trained using machine learning (ML) approaches to adapt to the new environment. Our proposed framework is effectively important for each type of system, as both systems require software development. The process of identifying and locating relevant software components can include attributes and properties associated with software components and applications such as design documents, implementation language, size, documentation, application domain, and version [23]. A person is capable of memorizing two units of information in working memory; this limited working memory allows for the comparison and organization of only two to four elements at any given time [24], so it is not possible for the developer to memorize everything. To reduce the cognitive burden, we have developed a platform that facilitates developers in finding their desired code. Robotics, IoT, and sensor-based systems are being developed rapidly and require building multiple components quickly. Our platform assists developers to locate the desired software components to accomplish the development of these systems. Furthermore, our platform can assist during the development phase of robotic, IoT, and sensor-based systems. Since these systems are developed and integrated rapidly and simultaneously; therefore, their codes must be safe and tested. IoT systems are in vogue and in demand and one needs to remember that they cannot be upgraded offline. In addition to this, one also needs to consider the fact that the developers do not have sufficient time to develop or upgrade the system. IoT systems are prone to change with each passing day. Thus, these systems are live and need coding support on an immediate basis. Therefore, to facilitate the development of these systems, our platform better recommends source code components. This platform not only facilitates the rapid development of many systems (such as IoT-based systems and robotic systems) but also allows developers to retrieve the desired component against the given query. The entered query searches for best-matched components, which are then utilized by developers during the development of systems. This platform uses ML techniques, for example, RankBoost algorithm [25] to learn ranking schema and measure context similarity, etc. This also allows the users to retrieve their desired components using one common platform. This platform provides a common means to search across various sites for the retrieval of required code components. Instead of searching across different sites, developers use this platform, which in turn reduces time and effort.

The most used languages for robotics include C, C++, and python. Software robot developers always wanted to save time, so instead of coding the robotics system from scratch, they look for the available code options. Our platform facilitates developers in this regard and allows them to enter their specifications. After getting the required code component, they integrate it into their system to check if the code works well with the existing code. This platform can be effectively utilized in all the areas where developers want to search code for the purpose of reusing it in their applications.

Source code is recommended using content-based filtering methods, where a description (query or requirement) for a specific software component is entered in the form of a query. This method uses keywords of the query which are matched with the corpus of source code components. We have employed a “RankBoost”, which is based on ML and has the ability to return the correct results because it is trained on the corpus. The RankBoost algorithms try to recommend the best fit component which is similar to the ones which the developer mentioned in the query. The evaluation results demonstrate that our approach returns accurate results with high precision.

Our platform recommends code components upon the query. Before using the retrieved code component the developer performs “Component Testing”. This testing checks each code component separately for its integration into the existing application. After the code component is successfully integrated, the developer performs “Integration Testing”. This testing determines whether or not the new integrated component with the existing application provides the desired functionality. If the search code does not work properly, then the developer looks for other code components in the ranked list to find the best match. In this research, our aim is to improve the retrieval of the code from existing resources. If there is any existing code for any specification (query) for which different pipelines are present, then our system can represent it as a code. However, two different recommendations can not be connected. In the future, one requirement specification can be converted into two sub-specifications for retrieval of source code. Regression testing is performed to ensure that the application’s current functionality is not affected by the change in any component. This testing confirms the product is compatible with any added features, problem repairs, or modifications to current features. Moreover, our platform uses a ranking schema. The ranking schema requires various performance features, also called ranking features (as mentioned in Table 1). These ranking features are utilized to compute the similarity between query and code snippets. For example, the idea behind including the popularity feature is that it recommends frequently used code components. Frequency of use depicts that these source components work as desired because these are tested whereas low popularity implies that code components fail to produce the desired results (i.e., fail during testing). The role of ranking schema is to place the pertinent code components in top-k results. However, it is challenging to ascertain the subjective construction (or configurations) of the ranking schema. Robotic systems may require different source code components in building a complete working system. For instance, it requires vision enhancement and filter code components which our platform recommends upon the entered specification (query). Our code is available at https://github.com/nazia-phd/implementation (accessed on 21 November 2022), this code can be utilized for study and further extension.

### 1.2. Research Contribution

This research paper has the following contributions:

- Proposed a framework that uses ML techniques to automatically recommend source code examples.
- We evaluated our framework using a dataset of 2500 code examples related to 50 queries. The evaluation results show that our proposed framework works effectively for source code components as it recommends relevant code examples for developers from existing schemas or online engines.
- A small prototype of the proposed framework is implemented and is qualitatively assessed using two experimental stages.

The rest of this paper is divided into sections, the detail of which is as follows: Section 2 consists of a literature review to elaborate on previous research. Section 3 includes the proposed methodology. Experimental evaluation is described in Section 4. Section 5 presented the conclusion of the study.

## 2. Related Work

Retrieval of the relevant component from the repository is one of the most difficult tasks. The software component assessment and evaluation depend on various criteria because of varying technical requirements, objectives, and business needs [26]. In many cases, component selection criteria might conflict with each other, and because of this, making the correct decision is quite difficult. Major problem during the component selection process include the varying component dependencies. It is possible that the match of a suitable component might not be found with available components in the software repositories. “Component-Based Software Engineering” (CBSE) facilities the construction of the system with the help of pre-existing software components [27]. This idea of CBSE is not new. It was visualized more than four decades ago by McIlroy [28]. McIlroy proposed the idea of commercial component production in line with the one that was found in other engineering fields. This section focuses on the literature review of the relevant material including various publications on matching properties and components. Most of the work in this domain is related to “Commercial-off-the-shelf” (COTS) and Quality of Service (QoS) but when it comes to component matching then QoS is mostly used.

The CBSE process also uses ontologies for the feature descriptions of software components, this eventually helps in the matching process. Numerous approaches have been proposed for component retrievals (e.g., [29,30]) that work on the modified forms of keyword-based and signature-matching techniques for component retrieval. In the light of research, besides our tool, other tools have been developed (e.g., [31]) to model software development. However, the drawback of these tools is that it employs few component features as shown in Table 2. For example, CodeBroker tool [31] utilizes both signature matching and free text techniques. Based on user comments that explain the intended functionality, this tool finds the relevant match. If the expected results are not obtained, the system considers the method signature following the comments. The contemporary open source code search engine, such as sourcerer [32] provides access to the source code but they normally fail to address the issue of the code’s structural information which can end in distracting the concern of stakeholders. A prototype named Ichi Tracker is developed by Inoue et al. [10] to give insight into code fragments. It provides an online search facility to detect code clones. This approach is effective as it takes code fragments as input and tracks the source code modified version and their origin, not only that potential violations of license are been traced. German et al. [33] inspected source code migration by keeping track of three different systems (Linux, FreeBSD, and OpenBSD). Their work also covered the legal implications of such migration of source code. They tracked the origin of reused code fragments using clone detection techniques. Their work revealed that migration of the code did occur between these systems. Moreover, the license terms were not violated during the code copying. Davies et al. [34] played a significant role in proposing a signature-based technique to find out the code entities’ origin. Furthermore, they discovered that their proposed technique can be employed in the reused libraries to identify security bugs. Kawamitsu et al. [35] further refined this work by proposing a technique that compares two repositories and automatically identifies the reuse of source code at the file level. His approach measures the resemblance between two source files in order to trace the original source file version.

Hang et al. [36] came up with framework-based applications. These applications use XML configuration files. The best thing about these files is their popularity in contemporary commercial applications. Unfortunately, the majority of the frameworks are either not documented well or are complicated, thus posing a massive challenge and complexity for developers regarding the correct use of a framework. To address this issue, another tool [36] is proposed that automatically suggests XML configuration codes by mining association rules and tree patterns. In order to accomplish this objective application repositories support developers in properly generating XML configurations during the production phase of software development. This tool recommends reusable XML snippets to help programmers to configure XML files [36].

Various systems have been proposed to automate the procedure of ranking source code snippets but unfortunately, the source code features (as mentioned in Table 1) are overlooked and are not measured properly by these systems. To address the said issue, Diamantopoulos et al. [37] proposed a code recommendation system called QualBoa that includes non-functional (quality attributes) and functional aspects of the code snippets. QualBoa provides a ranking of the retrieved code components not only on the basis of functional parameters of query but code reusability scores are also computed on the set threshold values of code metrics. The QualBoa evaluation shows that it is efficient to search and it recommends reusable code components [37]. Source code recommendation tools and approaches, as shown in Table 2, facilitate locating the desired code component that developers can integrate into their current systems. However, unfortunately, these recommendation systems do not properly employ the offered and desired properties of source code components. Our proposed approach considers various features of source code for ranking. It can be observed in Table 2, many strategies used a single feature or used heuristics to integrate features for the ranking of results. Second, the learning-to-rank approach is used for many software tasks, such as bug identification [38], source code fault detection [39], feature location, and traceability [40]. We implemented this approach because this can be robust and applied to source code snippets as well. Section 3 demonstrates the complete methodology of our proposed approach.

**Table 2:** Ranking approaches for source code.

Tool/ Approach	Strength	Weakness
Google Code Search and Ohloh [41,42]	Results are ranked based on textual similarity.	Uses only one feature which is textual similarity.
Sourcerer [29]	Uses the basic notation of CodeRank, which only extracts structural information.	Only focus on structural information of source code.
PARSEWeb [4]	Uses the frequency and length of MIS (method-invocation sequences) to rank the final result.	Uses MIS feature during the ranking phase.
Exemplar [43]	Uses three ranking schemes WOS (word occurrences schema), DCS (dataflow connection schema), and RAS (relevant API calls schema) to rank the application.	This tool ranks the applications, not the source code snippets.
Semantic Code Search [44]	The comparable code snippets that follow the call sequences extrapolated from code snippets determine the ranking.	Uses a call sequence, which is the only feature used for the ranking code snippets.
Pattern-based Approach [45]	This approach considers popularity to rank the working code examples.	Popularity is the only feature that contributed to the final ranking.
QualBoa [37]	This tool incorporates functional and quality attributes	Ranking components based on the functional score

## 3. Research Methodology

**Figure 1:**
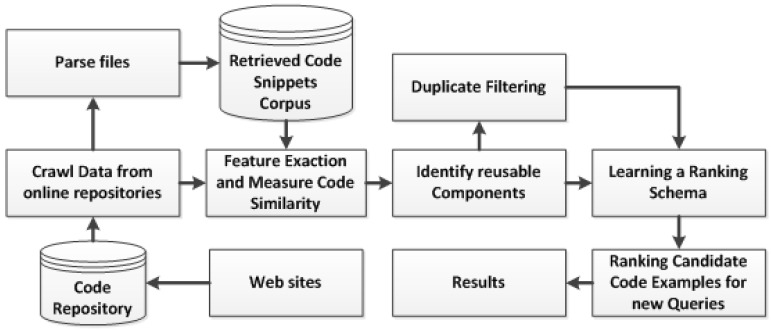
Proposed System Architecture.

The general method of our approach is detailed in this section. Figure 1 depicts the approach for the proposed solution. The fundamental concept is to develop a coding system for efficient component retrieval. The proposed methodology is described in full below:

### 3.1. Process Description

The overall process is divided into four stages: (1) selecting the language for code extraction; (2) crawling code components; (3) extracting features; (4) identifying reusable components; (5) raking schema learning; and (6) candidate code ranking for new queries. The ranking process takes place at run-time when a developer enters a query. The query is formulated when a user selects options from GUIs. The requested code snippets are then retrieved from the corpus. The retrieval process requires class or method names. The cosine similarity is computed among the source code and a query. Ultimately, the system chooses the code snippets as candidates on the basis of similarity and then the system displays the ranked list of selected candidates.

Selecting language: To retrieve code examples for queries the system provides the user interface to select the desired language to crawl the code.

Crawling Software Repositories/Projects: Reusable source code corpus is built to retrieve appropriate code examples for queries. The crawler looks for the project titles “Asp.net”, “PHP”, “C#”, “JavaScript”, “HTML” and “Python”. The crawled code snippets are downloaded to build the source code corpus. Eclipse JDT Java syntax parser is employed for extracting code snippets for all methods described in the source code file with the “java” extension.

Earlier work on extracting code snippets [44,45] recommends Java source code. Different parsers for “Asp.net”, “PHP”, “C#”, “HTML”, and “Python” can also be used to extract source codes of various languages. Table 3 shows the summary of the corpus that is used in our research. Our repository/corpus contains 585 projects in total. It contains 65,491 java files, 360,162 example codes, and 3,866,351 LOC of the code snippets.

Extracting Features: Each candidate source code is represented as a vector ($V_{s}$) and feature values are included in this vector which is obtained from code examples. $V_{s}=\left(f_{1},\ldots ,f_{i},\ldots ,f_{n}\right)$ is the set of features where $f_{i}$ denotes the $i^{th}$ feature value whereas *n* represents the total number of features.

Ranking Schema Learning Process: The proposed approach systematically uses the training data for the training of ranking schema. Queries and their given candidates’ results are included in the training set. We represent training data in the form of a set of triplets $\left(q,\;r,V_{s}\right)$. The query is denoted by *q* and *r* is the relationship between a code example and a query (*r*). RankBoost [25] algorithm is used to train the schema for ranking code snippets.

Code Ranking: A search query is entered and the ranking algorithm computes the candidates’ code fragment score. The computed score depicts the percentage of similarity among code candidates and a query. The ranking of source codes is performed on the basis of computed scores in descending order. Source code snippets that are ranked higher are considered more pertinent to the query which denotes that these source code examples are considered more useful than the ones that are ranked lower.

Extracting Features: For training the ranking schema, various features of code examples are required in our process. Twelve features are selected that have already been used in previous research [4,43,45]. Features are divided into 4 categories: context, popularity, similarity, and code metrics. Moreover, features are classified into two categories which are query-independent and query-dependent features [46]. Features that are relevant with respect to the queries are termed as query-dependent features whereas traits of source code examples, irrespective of the queries, are termed as query-independent features.

#### 3.1.1. Similarity Measure for Textual Information

The similarity of textual information is an essential feature for establishing the relevancy between query and candidate examples [4,47,48]. The vector space model (VSM) [49] is used to calculate the relevancy between candidate examples and queries. Relevancy between code components and queries is represented by weights that are stored in a vector. Later, the weight of each term is computed with the help of inverse document frequency (tf(termfrequency)−idf(inversedocumentfrequency)) and a weighting schema is generated for each term [50]. Weighting schema is used to depict the significance of the term in a particular collection of documents. The weights are computed using Equation (1).

(1) $$W_{t,d}=nf_{t,d}\times idf_{t},$$

where $nf_{t,d}=0.5+\frac{0.5\times tf_{t,d}}{{max}_{t\in d}\;\;tf_{t,d}}$, $idf_{t}=\frac{N}{df_{t}}$.

Manning et al. [50] explain the process of similarity computation for text. He describes different terminologies used in Equation (1). tf is the term frequency and $tf_{t,d}$ is the frequency of terms (*t*) that appears in document (*d*). Document (*d*) in our case refers to a query or code snippet. df is the document frequency and $df_{t}$ defines the number of times a term appears in several documents. $idf_{t}$ is the inverse document frequency obtained when dividing *N* (the total number of documents) by the $df_{t}$ (represents the number of documents in which term (*t*) occurs). Later, the textual similarity is measured using Equation (2).

(2) $$textualSim\left(V_{q},V_{c}\right)=\frac{V_{q}^{T}V_{c}}{|V_{q}\left|\right|V_{c}|}.$$

In Equation (2), *T* represents the transpose operator. The candidate code vector is denoted by $V_{c}$ and $V_{q}$ is referred to as a query vector.

The time complexity is O(n), which means that it increases linearly as the number of input queries increases whereas the space complexity can vary depending on input size and therefore it cannot be less than O(n) for the size *n*.

#### 3.1.2. Popularity

The latest research on code recommendation [4,51] uses an acceptance rate that distinguishes the candidate’s answers with the popularity rate [52]. Popularity shows the proximity of a source code with the frequent patterns that are appeared in a corpus of the code snippets. The fundamental basis of selecting a source code snippet is to ensure its relevancy to the frequent pattern in the corpus. Pattern popularity of the code example is independent of the query and is computed by making use of the frequency [4] or the feature of probability [53].

Popularity is measured using frequency and for this, we have employed the same procedure as given by Keivanloo et al.’s study [45]. The method call sequences are termed as usage patterns that are mostly used for applying rational functionality. The usage of pattern frequency depicts how many times a method calls work. A usage pattern occurrence of a code example is considered the popularity measure.

The code example’s usage pattern identification is done by extracting the sequence of method calls for each source code snippet available in the corpus and then mining techniques of frequent items are applied to analyze the number of method calls for the usage of patterns [54]. We determine the similar usage pattern of the method calls by calculating the cosine similarity. The popularity is measured by using the frequency; it can be computed by employing a probability-based process given by [53].

Extracted call sequences from the corpus are separated into two successive sets of method calls. For instance, method call order in a source code is labeled as $S_{m}=m_{1},m_{2},\ldots ,m_{n}$, *m* denoted the method and *n* shows the frequency of method calls in the source code snippets. Later, the ordered sequence calls are separated into the pairs of method calls $P=p_{1},p_{2},p_{i}\ldots ,p_{\left(n-1\right)}$, where $p_{i}$ signifies the method called pair $\left(m_{i},m_{i+1}\right);\left(m_{i},m_{i+1}\right)$; the $m_{i}$ method is called prior to $m_{i+1}$. In the corpus, all sequenced method calls are arranged in pair structures, we calculate the probability of the method-called pair pi as $P(p_{i})=1/N$, where *N* counts the frequency of calls for method pairs and $m_{i}$ is the method called prior to another method in the corpus. Hence, the frequency of feature occurrences for code snippets is computed using Equation (3) [53].

(3) $$probability=\Pi_{i=1}^{n-1}P\left(p_{i}\right).$$

Invocation of the method in the code example is represented by *n* and the method call pair probability is denoted by $P(p_{i})$.

The time complexity is O(n), which means that it increases linearly as the input queries increase, whereas the space complexity can vary depending on the input size and, therefore, it cannot be less than O(n) for the size *n*.

#### 3.1.3. Code Metrics

Previous studies employed four code metrics for code search and code quality estimation [51]. Query-independent features are grouped into a set, which is termed as code metrics. Table 1 abridges the metrics that we used throughout the approach. Buse et al. [51] specified that the total number of LOC (line of codes) and the average number of associated identifiers with each LOC are used to determine the reliability of the code. Code reliability is the quality metric that is measured using the average number of identifiers on each line of the code. The call sequence length depicts the total number of method calls in a source code’s example call sequence. The involvement of the code comments can be judged by the comment code ratio in the code examples. The complexity of source codes can be measured by the page ranking, and fan-in and fan-out.

Fan-in is referred to as the total number of calls for a particular source code. The number of times a source code is called by other codes is termed fan-out. The algorithm for page rank works to determine the importance of the source code snippets by calculating the total number of links of a specific code example. The fundamental idea is that more important source codes gain more links from other source codes. Page rank is an important metric and it can be for code search [48], code snippets graph is built on the basis of the number of times a particular code is called. In the graphic representation, if code fragments *A* call code fragment *B*, then a link relation is formed between both of them. Later, the value of page rank is computed for each code fragment separately by using the graph R package (“igraph”). Assume, code fragments $C_{1},C_{2},\ldots ,C_{n}$, call code fragment *C*, and N(E) depict the total code fragments that are called by code fragment *E*. Later, the page rank is computed for each code snippet (E) using Equation (4) [55].

(4) $$PR\left(E\right)=\left(1-d\right)+d(PR\left(E_{1}\right)/N\left(E_{1}\right)+...+\left(PR\left(E_{n}\right)/N\left(E_{n}\right)\right)).$$

The damping factor is denoted by *d* and we take its value equal to 0.85. [55].

#### 3.1.4. Measure Context Similarity

Context similarity is used to measure the commonalities among query contexts and source code snippets [56]. Previous studies [57] observed that the success of the code search is accelerated by employing contextual features. The method signature of the code and a method body’s signature are used when a query is raised, these two types of signature help in predicting the query and source code context. The signature of the method is tokenized when a query (q) is raised using camelCase splitting and tokenized set is denoted as $S_{q}$. Likewise, code snippets’ method signatures are tokenized to formulate tokenized term set which is represented as $S_{c}$. Later, similarity can be calculated among code examples and a query with the help of the Jaccard index [58] which can be determined using Equation (5),

(5) $$contextSim\left(S_{q},S_{c}\right)=\frac{\left(S_{q}\cap S_{c}\right)}{\left(S_{q}\cup S_{c}\right)},$$

where $S_{q}$ is the set involving the tokenized terms from the query and $S_{c}$ is the set involving the tokenized terms of the code example.

**Figure 2:**
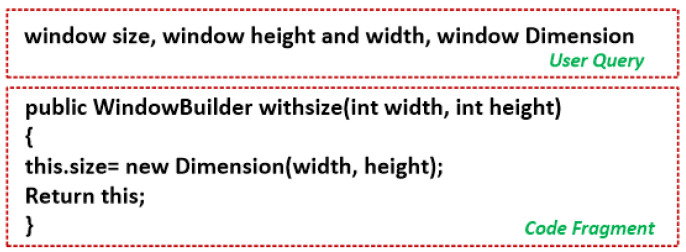
User query with code fragment.

For example, consider Figure 2 which shows the user query and its corresponding code snippets. The user enters a query, “window size, window height and width, window Dimension”. The class “WindowBuilder” invokes the “withsize” method which computes the window dimension. The tokenized form of the code snippet $\left(S_{c}\right)$ is: {“Window”, “Builder”, “with”, “size”, “width”, “height”} and the tokenized form of the user query $\left(S_{q}\right)$ is: {“Window”, “size”, “width”, “height”, “dimension”}. $\left(S_{q}\cap S_{c}\right)=\left\{4\right\}$ and $\left(S_{q}\cup S_{c}\right)=\left\{7\right\}$. So, according to Equation (5) the contextual similarity between the user query and code snippet is = 5/6 = 0.83.

The time complexity, in this case, is O(n), which means that it increases linearly as the context similarity is computed for several input queries whereas the space complexity can vary depending on the size of the query for context similarity and, therefore, it cannot be less than O(n) for size *n*.

**Figure 3:**
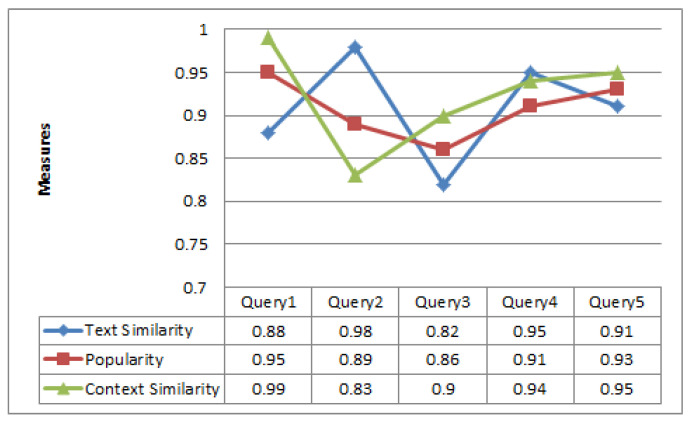
Queries returned results for various component features.

Figure 3 summarizes the results of accuracy achieved by using each feature as listed in Table 1. As we can observe the resultant values for each measure is satisfactory which means the error ratio is not too high. Therefore, we can conclude that the selected features can be used for ranking.

### 3.2. Training of a Ranking Schema

Freund et al. [25] proposed a RankBoost algorithm for ranking. This algorithm is used to train the schema, which is then used to rank candidate components. This ranking algorithm only takes a few minutes to train the schema because of its efficiency in training data. A brief description of the RankBoost algorithm is explained in this section.

Training data served as input to the ranking algorithm. The training set involves the code examples that are relevant to a particular set of queries. A vector $\left(q,\;r,V_{c}\right)$ represents each code example. *q* represents the id of the query whereas *r* represents the relevancy of the code and $V_{c}$ is the code snippets feature vector. ϕ is the feedback function that uses information on code examples and information on the training phase. $\left(c_{0},\;c_{1}\right)$ represents the pairs of the code snippets, $\phi (c_{0},\;c_{1})$ in the training phase signify the difference between the tag relevancy of $c_{0}$ and $c_{1}$. If $\phi (c_{0},\;c_{1})>0$ then the code example $c_{1}$ is tagged with higher relevance than $c_{0}$ and $\phi (c_{0},\;c_{1})<0$ implies the reverse. If $\phi (c_{0},\;c_{1})=0$ then it signifies that there exists no relevancy between $c_{0}$ and $c_{1}$.

RankBoost algorithm objective is to determine the decisive raking *H* which is akin to the feedback function ϕ. The purpose is to increase the similarity rate and for this more attention is towards decreasing the frequency of unordered instances of code pairs during the final ranking. Suppose a function $D\left(c_{0},\;c_{1}\right)=x*max0,\phi (c_{0},\;c_{1})$.This function sets all negative feature’s value of ϕ to zero. *x* denotes the positive constant whose value is set such that $\sum_{\left(c_{0},\;c_{1}\right)}D\left(c_{0},\;c_{1}\right)=1$. The code pair is considered important if $(c_{0},\;c_{1})>0$. Final raking (*H*) is determined by altering the RankBoost algorithm, which basically computes the weights in a way that minimizes the unordered pairs of code fragments, which is termed as the ranking loss [25] and is computed using Equation (6),

(6) $$loss=\sum_{\left(c_{0},\;c_{1}\right)}D\left(c_{0},\;c_{1}\right)\left|H\left(c_{1}\right)\leq H\left(c_{0}\right)\right|,$$

where $|H\left(c_{1}\right)\leq H\left(c_{0}\right)|$ is 1, if $H\left(c_{1}\right)\leq H\left(c_{0}\right)$ and 0 otherwise.

The learning process details of the RankBoot algorithm for approaching the final ranking *H* is shown in Algorithm 1 [25]. RankBoost works in rounds. The ranking function $f_{t}$ in each round is produced based on the raking feature list as provided in Table 1. Meanwhile, the values of $D_{t}\left(c_{0},\;c_{1}\right)$ are maintained for all pairs of code examples, and these values highlight different sections of training data. The ranking function $f_{t}$ is utilized by the ranking algorithm to modify the $D_{t}$ value in each round as shown in Equation (7),

(7) $$D_{t+1}\left(c_{0},\;c_{1}\right)=D_{t}\left(c_{0},\;c_{1}\right)exp\left(\alpha_{t}\left(f_{t}\left(c_{0}\right)-f_{t}\left(c_{1}\right)\right)\right),$$

where the $D_{t}\left(c_{0},\;c_{1}\right)$ value is maintained for every code fragment pair in each iteration *t*. The ranking function is denoted by $f_{t}$, which is produced at iteration *t*, and αt is the standard factor for $f_{t}$, and αt>0 [25]. Based on Equation (7), the algorithm assigns $c_{0}$ with the highest rank value than $c_{1}$. $D_{t+1}\left(c_{0},\;c_{1}\right)$ will minimize if the ranking function $f_{t}$ gives an accurate ranking $\left(f_{t}\left(c_{1}\right)>f_{t}\left(c_{0}\right)\right)$ and maximize otherwise. Therefore, $D_{t+1}$ tends to emphasize the disordered code examples. The loss of the final ranking loss (*H*) is calculated [25] using $\left(H\right)\leq \Pi_{\left(t=1\right)}^{T}D_{\left(t+1\right)}$. For ranking schema (H), we have to decrease $D_{t+1}$ in order to decrease the loss function. Freund et al. [25] work shows that $D_{\left(t+1\right)}$ is decreased when

(8) $$\begin{array}{cc} \alpha_{t}=\frac{1}{2}\ln\left(\left(1+l_{t}\right)/\left(1-l_{t}\right)\right),l_{t}= & \sum_{\left(c_{0},c_{1}\right)}[D\left(c_{0},c_{1}\right)\\ & (f_{t}\left(c_{0}\right)-f_{t}\left(c_{1}\right)))]. \end{array}$$

In each round *t*, $D_{t}\left(c_{0},\;c_{1}\right))$ denotes the sustained value of the code fragment; $f_{t}$ represents the ranking function, which is computed at each iteration *t*; and $\alpha_{t}$ is an important factor for $f_{t}$, and $\alpha_{t}>0$ [25].

During this approach, the aim is to reduce the rate of loss function for the ranking (*H*). Equation (8) achieves the value of variable $\alpha_{t}$ for the ranking function $f_{t}$. $H\left(c\right)=\sum_{\left(t=1\right)}^{T}\left[\alpha_{t}f_{t}\left(c\right)\right]$ denotes the weighted sum of ranked features during the final raking process. The H(c) obtained the optimal results with respect to the ranking loss function.
**Algorithm 1:** The learning process details of the RankBoot algorithm for approaching the final ranking *H*.Require: Initial values of (D $c_{0}$, $c_{1}$) over each code example pair in the training data.1: Initialize: $D_{1}\left(c_{0},c_{1}\right)=D\left(c_{0},c_{1}\right)$2: for t = 1, …, T (T = 12) do3: Build ranking function f t (c) based on the ranking feature.4: Choose $\alpha_{t}$ using Equation (8).5: Update: $D_{t+1}\left(c_{0},c_{1}\right)=D_{t}\left(c_{0},c_{1}\right)exp\left(t\left(f_{t}\left(c_{0}\right)-f_{t}\left(c_{1}\right)\right)\right)$6: end for7: Output the final ranking $H\left(c\right)=\sum_{t=1}^{T}\alpha_{t}f_{t}\left(c\right)$

## 4. Experimental Evaluations

The experiments were conducted to measure and evaluate the applicability, efficiency, effectiveness, and usefulness of the proposed approach. We have divided the experiments into two stages. Stage A entails searching for and obtaining various components based on a set of characteristics, whereas Stage B entails investigating certain reusable activities. Stage B is critical as it is an elaboration of our approach because it provides an in-depth and extended version and involves reuse development activities facilitated by our tool. The feedback from both experimental stages is considered to improve the proposed approach. Evaluation metrics and comparable baselines are utilized in Experimental Stage C to assess the correctness of the proposed strategy.

### 4.1. Experimental Stage A

**Figure 4:**
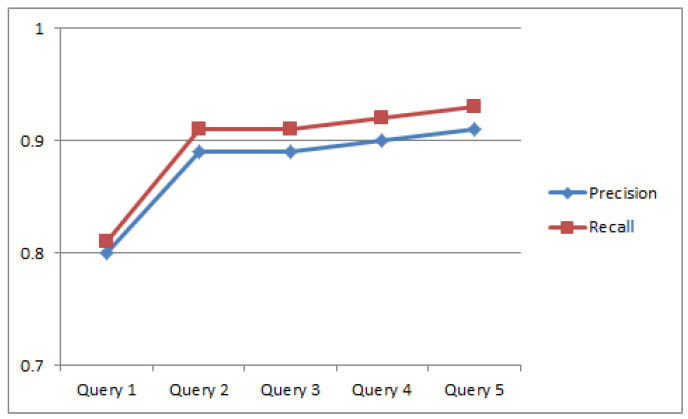
Precision and recall.

The first step is to train the ranking schema and for this, we used the RankBoost algorithm proposed by Freund et al. [25]. The algorithm is effective as it accelerates the training process and requires a short time based on our proposed reuse CBSE framework. The proposed approach used semi-formal natural language structures and also concentrates on the ranking schema. The main aim is to evaluate the effectiveness, efficiency, usefulness, and applicability of the framework. Hence, the experiment was conducted to answer the following research questions related to our proposed framework. (Q1) Is the process convenient and simple to find suitable components? The stated question basically concentrates on the level of understandability, efficiency, and better applicability of reusability, functional, and non-functional properties to find and target the candidate components. (Q2) Is the process of locating a suitable component complete or not? This question defines the availability of information and whether the information is enough to facilitate the framework or provide complete information to the user. Completeness is a characteristic that cannot be easily quantified. However, for the sake of this evaluation, we consider that the completeness shows the extent to which the suggested process facilitates the profiling of all the areas producing information regarding the components. (Q3) How accurately does the system recommend the components? The accuracies of the returned results are computed using evaluation metrics (recall and precision). Recall and precision measures are applied to rectify the correctness of the results. We have used 50 benchmarked queries [59] for the evaluation of our approach. We randomly selected five queries and calculate precision and recall. The average recall and precision of five queries are 0.878 and 0.896, respectively, as presented in Figure 4.

(Q4) What is the process effectiveness in terms of time/effort when the user is trying to search and locate the desired component? This question pertains to the quality of the result in terms of efficiency and effort that is required. It also refers to the satisfaction level of the user. We randomly crawled around 100 synthetic components and then these product components were examined by the practitioners to propose corrections so that realistic cases can be handled. We divided the created components into seven categories; GUI Widgets (Wallworks (15), Login (10), Address Book (10), Calendar (10), Calculator (10), Clock (10), Background/Fonts Style (10)), Window Style (15), and Task Manage (10). Synthetic components have multiple instances for each category to distinguish the attributes, such as OS, protocols/standards, programming language, openness, documentation, and also the indicators of the performance.

The EBNF (Extended Backus–Naur Form) identity of each component was generated, which was followed by its transformation into the instance of the ontology of the component tree. The language was chosen by the user and the chosen language was made to function 10 various searches with the help of a simple form. In EBNF, we also transformed the query information and then moved to the ontology tree instance, which was also generated Each instance of the search tree was then checked against the component instances available in the repository. Two classic metrics recall and precision is computed for the evaluation of retrieved components. The retrieved components satisfy the functional constraint and other features. Candidate components similarity is calculated and the system ranks the candidate components that fulfill the requirements.

Table 4 gives a detailed view of the experimental procedure. The components’ functionality is shown in the first column while the component properties are in the column on the right side and the five candidates (retrieved component) are enlisted in the middle. The precision and recall of five queries are shown in the lower part of Table 4. Query 5 retrieved the desired candidate because it assures the coverage of maximum search criteria as compared with other components with high precision and recall. We executed the procedure 10 times for every component category and then results are calculated and assessed qualitatively. Four questions are formulated for the qualitative assessment of the approach. In the end, the participants were requested to evaluate the approach and for this, a five-point scale is used where 1 represents the lowest rating and 5 represents the highest rating.

**Figure 5:**
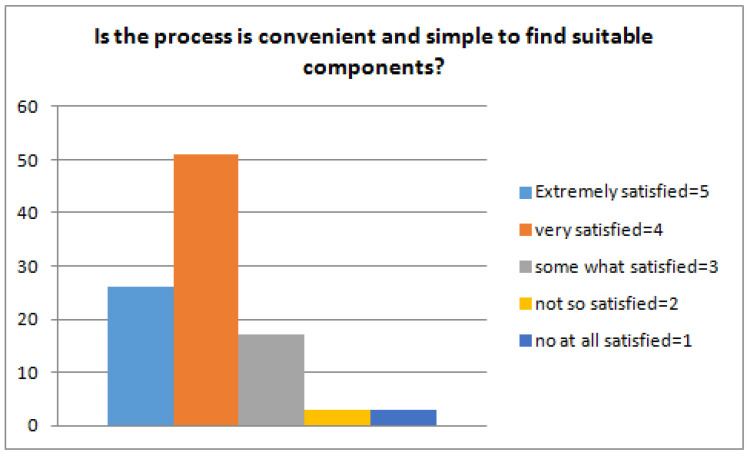
Process suitability to retrieve components.

**Figure 6:**
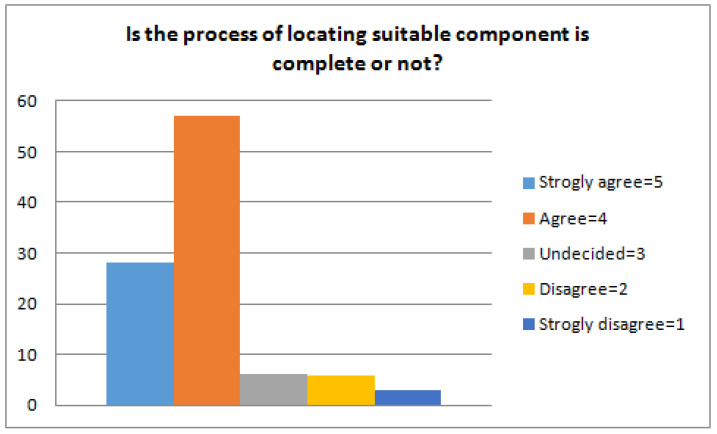
Process completion for retrieving components.

**Figure 7:**
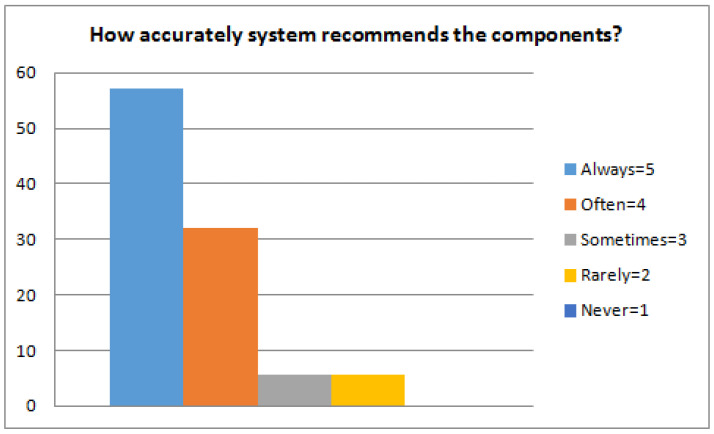
Accuracy of recommended components.

**Figure 8:**
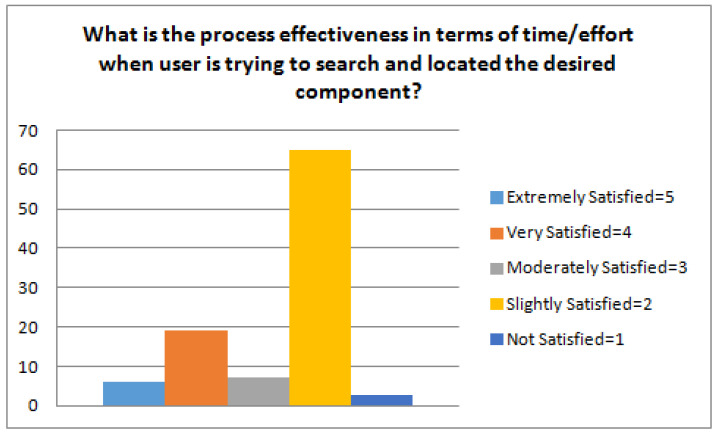
Component retrieval process effectiveness.

The experimental results are summed up in the respective questions given below:

(Q1) Is the process convenient and simple to find suitable components? The audience agreed that the approach is easy if trained once. As we can see in Figure 5, the majority of the respondents rate it 4 (high). The training process is not too intricate to follow and the effort it requires is acceptable. Searching for desired code fragments is much easier and facilitated by our tool. After searching a few times, they felt comfortable and barely faced any issues throughout the process.

(Q2) Is the process of locating a suitable component complete or not? Completeness is the characteristic that arouses some questions. As can be observed in Figure 6, initially, the developers rate this as 4, which is considerably high, whereas practitioners rate it 2, which is considered low. Practitioners are more experienced and they suggest that the proposed approach can be improved more. We conducted a series of discussions to explain the process and nature of the profiling scheme to practitioners. Apart from that, great emphasis was placed on how new properties may be added to fulfill other requirements and practitioners were told to rank the question once again. After that, practitioners realized that the present profile design can be improved. Our approach is extended by incorporating new features and source code components in the corpus. Hence, the practitioners reached the point that the method was quite flexible for an extension. Therefore, it was ranked with a median value of 4, which is considered High.

(Q3) How accurately does the system recommend the components? The system recommends components that are deemed useful and are considered to be candidates. As can be seen in Figure 7, the majority of respondents rated the system’s accuracy as high (5). The system ranked the candidates’ code components on the basis of the selected options. It is observed that the systems recommend the best candidates because the returned results satisfy the threshold values of certain properties. The recommended component exhibits a satisfactory balance between the statistical characteristics and the rating. The user chooses the best component based on the ranked results and matched properties. Table 4 shows the optimal results for the most suitable component. The optimal results are highlighted in bold and italics. It can be seen that component 4 is considered the best candidate because it has the maximum optimal value. The retrieved components are accurate in relation to the given query (precision). High precision shows that most of the retrieved components are pertinent. High recall shows that some of the relevant components are left, i.e., they are not retrieved.

(Q4) What is the process effectiveness in terms of time/effort when a user is trying to search and locate the desired component? As shown in Figure 8, effort and the amount of time put to determine the suitable components was limited; hence, it was ranked 2, i.e., low. For the sake of giving back the right components to the user, automation is considered in order to return the most suitable component and acknowledge a user within acceptable time limits.

Experimental outcomes, specifically question 3, clear the fact that a limited amount of alterations/modifications can be made to achieve better performance. Our approach helps users to retrieve the candidate components depending on their requirements.

### 4.2. Experimental Stage B

The second stage of the experiment was conducted which was similar to the first stage of experimental design. In order to evaluate the scalability of the proposed approach users were asked to develop a small application by searching code components using our tool. Experimental stage 2 mainly aims to achieve two objectives.

Objective 1 is to extend and enhance the use of the proposed approach. In order to achieve this objective, we have prepared an experimental setup for the user. The experimental setup is easy as it involves the development of complex tasks. The developer searches for the required code component and uses it in the application. During the development of the application, the developer has to check the compatibility of software components.

Objective 2 is to inspect the efficiency and scalability of the approach. The approach was extended at the recommendation level by adding a module that identifies the compatibilities issues (i.e., operating system and programming language); afterward, the system recommends the most suitable ranked components that provide developers with cost-effective solutions in terms of time and effort. The system gives such types of recommendations only in case of incompatibilities that are related to the characteristics of the code snippets. The second objective can be attained by extending the functionality of the approach that offers users a mechanism to define the desired properties of code components with varying degrees of importance. A weighted scheme method is utilized to assign the value to each attribute of the code component; for this, we are using a triplet scale whose values range from 1 to 3, which represent low, medium, and high, respectively.

**Figure 9:**
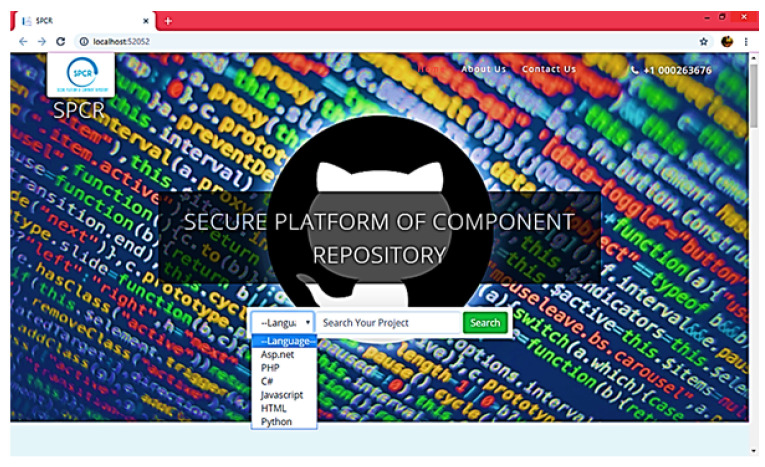
Select Language.

**Figure 10:**
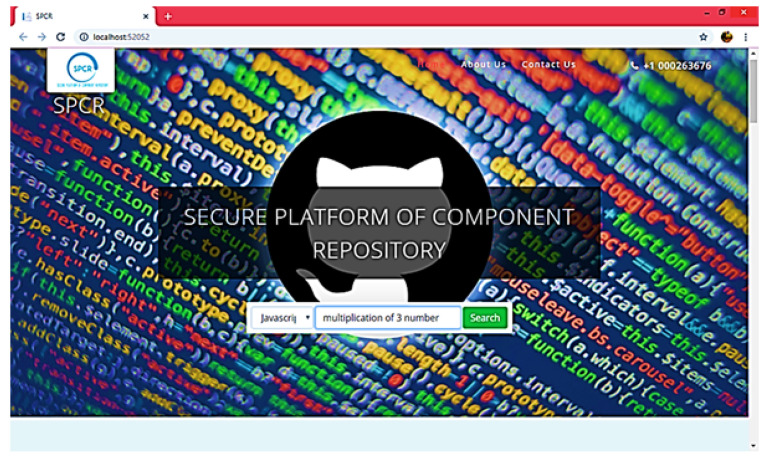
Select component/function for Search.

**Figure 11:**
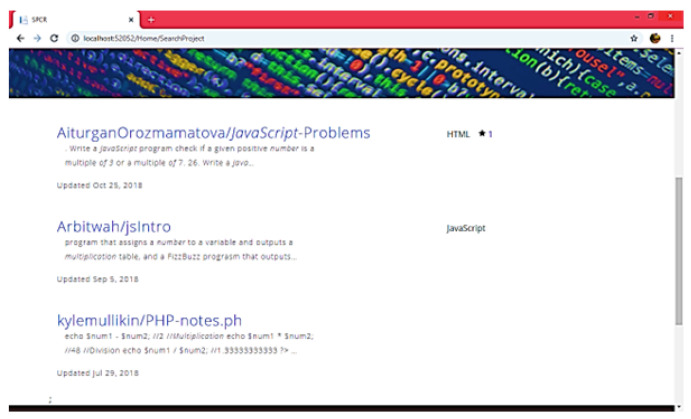
Crawled Results.

A component corpus was created and 12 features were studied during the generation of components with varying properties as defined in Table 1. The user was given a randomly selected list of various features that guided the user to select the suitable components for each scenario. The system provides a user-friendly interface where users can easily set the component feature priorities and then retrieve the final component that is considered most appropriate, as shown in Figure 9, Figure 10 and Figure 11, where each user is allowed to access the server’s corpus with the help of simple GUIs to find and retrieve the components. Search attributes are provided in the form of pull-down menus and drop-down lists. Later, the definitions of these properties were transformed automatically into ontologies and corresponding EBNF forms with minimal effort at the user end.

The experiments of stage B spanned a time of one week. In the end, the experiences gained from the experiment were shared with the audience and the framework parameters were rated again. Experimental result examinations were performed in a similar way as presented in the previous stage: (Q1) Is the process convenient and simple enough to find suitable components? The received responses depict that the approach is straightforward and easy. The respondents rated this question considerably high (i.e., 4). The authenticity of the results was challenged when it was realized that, initially, the developer was unable to work with the tool. However, the larger application size and wide scope of the approach decreased this challenge. (Q2) Is the process of locating the suitable component complete or not? The extension of the proposed approach to accommodate new measures that define component properties was positively evaluated by the participants involved in the experiment. The process completeness is rated as 4, which is quite high. During participant interaction with our tool, a comment was received, which stated that the experimental stage 2 provides a more realistic working environment in terms of complexity. The reason is the extended version of the system incorporates more properties, which is why the completeness level is enhanced. (Q3) How accurately does the system recommended components? Code component accuracy is quite low as it is rated 2 and this is because the nature of the code snippets is different and the list of attributes that each participant provides also varies. For instance, in a few scenarios, the component properties were not aligned appropriately. For example, the application component that shows the list of products requires less time to respond as compared with the shopping cart component, which requires more response time. Hence, the audience under study talks about the components’ properties and realistic production within the underlying framework. However, the response was positive and everyone agreed that this glitch does not affect the experimental results because examining the applicability of the method was more complex. Moreover, our tool utilizes the weighted ranking scheme to achieve results with better accuracy. The weighted ranking scheme allows the users to minimize the number of matches, specifically when components are very close to each other in terms of set properties. Furthermore, the working of the ranking scheme was effective in most of the scenarios and only a few participants indicated that this weighted ranking scheme is not beneficial. In a few scenarios, the incompatibility of the programming platforms hinders the components’ composition (the recommended component not always being integrated into the set environment) but a blend of the above-stated scenarios was considered better to make the approach more compatible. A wrapper is developed easily to manage the incompatibility issues, which recommends better results. However, the limitation of our tool is how its applicability in terms of increasing the component selection support for users. (Q4) Is the process effective in terms of time and effort when the user is searching for and locating the desired component? Overall, the efficiency is rated high, which is 4. The rating is quite high because the search procedure is versatile. Hence, from the results, it can be observed that the complexity of the approach is not affected by the size of the application being developed. The development time taken to target all component instances for each case may vary.

### 4.3. Experimental Stage C

Stage C discusses the evaluation procedure to measure the accuracy of the proposed approach. In Section 4.4, different evaluation metrics are explained whereas in Section 4.5, we have different baselines.

### 4.4. Automatic Evaluation Metrics

Many application domains and previous studies [60,61,62,63] used various evaluation metrics depending on the recommender systems. To determine how effectively a recommender system performs, we used the following evaluation measures.

■ R@k: This metric is commonly used to evaluate whether the proposed approach can retrieve the correct code component in the top-k returned results. Equation (9) is used to calculate the value of this metric.
  (9) $$\begin{array}{c} R@k=\frac{1}{\left|Q\right|}\sum_{q=1}^{\left|Q\right|}\delta \left(Frank_{q}\leq k\right), \end{array}$$
  where δ(.) represents the ranking of correct code components against a given specification (query(*q*)) and *Q* represents the set containing all queries. If the relevant code component cannot be found in the top-k results, the frank function ($\delta (Frank_{q}\leq k)$) returns 0, otherwise, it returns 1. The better code retrieval performance is shown by a higher rank (R@k) value.
■ MRR: “Mean Reciprocal Rank” is represented by MRR. This metric is frequently used for the code component retrieval task. It computes the mean reciprocal ranks of the correct code components of query set Q. Equation (10) is used to measure the MRR.
  (10) $$\begin{array}{c} MRR=\frac{1}{\left|Q\right|}\sum_{q=1}^{\left|Q\right|}\frac{1}{Frank_{q}}. \end{array}$$
■ NDCG: It is the “Normalized Discounted Cumulative Gain” denoted by NDCG. This approach is frequently used to assess the value of a set of search results. This is computed by dividing “discounted cumulative gain” (DCG) and “deal deduct cumulative gain” (IDCG). Equations (11) and (12) are used to compute NDCG.
  (11) $$\begin{array}{c} DCG=\sum_{i=1}^{p}\frac{2^{rel_{i}}-1}{log_{2}\left(i+1\right)}, \end{array}$$
  (12) $$\begin{array}{c} IDCG_{p}=\sum_{i=1}^{\left|REL\right|}\frac{2^{rel_{i}}-1}{log_{2},\left(i+1\right)} \end{array}$$
  where *p* is the rank position, $rel_{i}$ is the graded relevance of the result at position *i*, and ${\left|REL\right|}_{p}$ is the list of relevant results up to position *p*. We set *p* equal to 10 for all experiments.

To summarize, the higher the values for the above automatic metrics, the better performance the approach achieves.

### 4.5. Comparison Baselines

To illustrate the effectiveness of our methodology, we chose seven baselines and contrasted them with our strategy. Below is a detailed summary of the baselines.

■ Google Code Search and Ohloh [41,42]: Ohloh and Code Search, similar to other search engines, retrieves code snippets and ranks them based on the query’s textual similarity.
■ Sourcerer [29]: It is an open-source search engine that implements the fundamental idea of CodeRank. Instead of using conventional keyword-based search, this engine extracts source code structural to perform the search.
■ PARSEWeb [4]: The PARSEWeb tool scans the local source code repository to extract different “method-invocation sequences” (MIS) and groups comparable MISs using a sequence post-processor. The retrieved MISs can be used to answer the supplied query. Results ranking is based on the frequency and length of MIS.
■ Exemplar [43]: This is a search engine that searches the relevant software projects based on the query. Three ranking techniques employed by Exemplar are WOS, DCS, and RAS. These raking schemes perform ranking to sort retrieved application list.
■ Semantic Code Search [44]: This “Semantic Code Search” approach retrieves similar code snippets that are ranked based on the call sequence extracted from code snippets.
■ Pattern-based Approach [45]: This approach uses three features—similarity, popularity, and line length—to identify working code examples.
■ QualBoa [37]: This is a recommendation system that retrieves source code components by considering both the functional and non-functional (quality aspect) requirements of the source code snippet. The ranking of source code snippets is performed based on reusability score and functional matching.

Table 5 summarizes the proposed approach’s consequences in line with baseline methods. Specifically, the first column indicates MRR. We can observe that the performance of various approaches varies depending on the type of features and ranking scheme. For example, our approach and QualBoa’s performance are best in terms of MRR, while the performances of the pattern-based approach and sourcerer are comparatively low. As the mentioned approaches do not employ the “Learning-to-Rank” approach, they may result in low MRR, R@10, and NDCG.

The results of our approach are presented in the last row. Our approach outperforms by a remarkable edge, specifying that our approach can return more relevant source code snippets. It is evident from Table 2 that the baseline approach mostly used a single feature with no “Learning-to-Rank” approach. Our approach incorporates various code snippet features along with “Learning-to-Rank”, which makes our approach more productive and precise in the code search. Finally, we can see that our approach outperforms in terms of overall performance measures (MRR, R@10, and NDCG).

## 5. Conclusions and Future Work

This research focuses explicitly on the topic of CBSE, particularly on the matching of a software component; its automatic search and retrieval issues are specifically addressed for the development of robotics, sensors, and IoT-based systems. The proposed approach facilitates and reduces the developer’s effort in code search by analyzing, assessing, and locating the most relevant available components. Our approach creates a ranking schema by employing the ranking algorithm. Twelve features of code components are considered for the training and testing phase. The training dataset is used to train the ranking schema. Source code components for new queries are ranked by the ranking schema. In light of the experimental results, we can conclude that our proposed approach is quite pragmatic and accurate. Apart from this, our approach is efficient and complete; it is handy to practice software reuse. The proposed approach is promising for a component recommendation but there exists ample room for enhancement and improvement.

Future work can be extended in many directions. First, the efficiency and applicability of the proposed framework can be validated through more detailed experimentation. Second, the component retrieval can be improved by employing evolutionary algorithms (optimization techniques) to automate the component search and retrieval process. Third, we used the rank-boost algorithm for ranking the retrieved candidate’s components, other ranking algorithms can be used to achieve better ranking results. Finally, a software tool is developed to support the proposed framework. The tool performance can be improved by employing additional capabilities of optimization techniques, EBNF editing, and the parsing process during the construction of the component profile. Further, the graphical and visual aspects of our prototype can be enhanced.

## Figures and Tables

**Table 1:** Component’s Features.

Category/Class	Feature	Description	Query Dependency
Similarity	Textual Similarity	Candidate code example and query likeness is measured using cosine similarity.	Yes
Popularity	Frequency	Code example method frequent occurrence of a specific in the corpus	No
	Probability	The frequency at which a method calls a code fragment. method	
Code Metrics	Line length	The candidate code length in terms of lines.	No
	Number of identifiers	The average amount of identifiers associated with each LOC of a candidate fragment	
	Length of call sequence	Number of times a candidate code component method is called	
	Code Comment ratio	The number of comments associated with candidates LOC	
	Page Ranking	A metric to compute significance of code snippets	
	Fan-in	The number of times a specific code snippet calls other unique code snippets	
	Fan-out	The number of times a specific code snippet is called by other code fragments	
	Cyclomatic complexity	Occurrences of decisions in a code component (for, while, etc.)	
Context	Context similarity	The Jaccard similarity measure is computed when a query is raised; it calculates the likeness between the method’s body and code example signature	Yes

**Table 3:** Corpus Summary.

Item #	Amount
Projects	585
Java Files	65,491
Code components	360,162
Code snippets LOC	3,866,351

**Table 4:** Candidates’ component evaluations.

Task	Query 1	Query 2	Query 3	Query 4	Query 5
Primary Input 1	Select Language	Select Language	Select Language	Select Language	Select Language
Primary Input 2	Select function to be retrieved	Select function to be retrieved	Select function to be retrieved	Select function to be retrieved	Select function to be retrieved
Response Time (sec)	10	12	8	8	9
Download history time(sec)	6	8	22	4	20
Memory utilization(KB)	2	3	4	1	2
Reliability (max)	90	95	92	93	90
Application domain	ANY	ANY	ANY	ANY	ANY
Operating systems	Windows	Windows	Windows	Windows	Windows
Precision	0.75	0.83	0.89	0.90	0.91
Recall	0.81	0.91	0.91	0.92	0.93

**Table 5:** Comparison baselines.

Tool/Approach	MRR	R@10	NDCG
Google Code Search and Ohloh [41,42]	57.81	66.10	69.01
Sourcerer [29]	66.01	69.02	68.01
PARSEWeb [4]	66.01	69.02	68.01
Exemplar [43]	62.05	71.02	69.50
Semantic Code Search [44]	67.40	79.02	70.08
Pattern-based Approach [45]	52.10	64.65	71.09
QualBoa [37]	67.00	81.50	75.09
Our approach	73.90	84.31	77.30

## Data Availability

Not applicable.

## Abbreviations

The following abbreviations are used in this manuscript:

MDPI	Multidisciplinary Digital Publishing Institute
DOAJ	Directory of Open Access Journals
TLA	three-letter acronym
LD	linear dichroism

## References

[1] Gharehyazie M., Ray B., Filkov V. Some from here, some from there: Cross-project code reuse in github. Proceedings of the 2017 IEEE/ACM 14th International Conference on Mining Software Repositories (MSR).

[2] Haefliger S., Von Krogh G., Spaeth S. (2008). Code reuse in open source software. Manag. Sci..

[3] Ponzanelli L., Bavota G., Di Penta M., Oliveto R., Lanza M. Mining StackOverflow to turn the IDE into a self-confident programming prompter. Proceedings of the 11th Working Conference on Mining Software Repositories.

[4] Thummalapenta S., Xie T. Parseweb: A programmer assistant for reusing open source code on the web. Proceedings of the Twenty-Second IEEE/ACM International Conference on Automated Software Engineering.

[5] Pastebin (2008). BWorld Robot Control Software. http://pastebin.com/.

[6] Discover Gists. https://gist.github.com/.

[7] (2009). codeshare. https://codeshare.io/.

[8] Reiss S.P. Semantics-based code search. Proceedings of the 31st International Conference on Software Engineering, IEEE Computer Society.

[9] Mockus A. Large-scale code reuse in open source software. Proceedings of the First International Workshop on Emerging Trends in FLOSS Research and Development (FLOSS’07: ICSE Workshops 2007).

[10] Inoue K., Sasaki Y., Xia P., Manabe Y. Where does this code come from and where does it go?–Integrated code history tracker for open source systems. Proceedings of the 34th International Conference on Software Engineering.

[11] Sadowski C., Stolee K.T., Elbaum S. How developers search for code: A case study. Proceedings of the 2015 10th Joint Meeting on Foundations of Software Engineering.

[12] Rosen C., Shihab E. (2016). What are mobile developers asking about? a large scale study using stack overflow. Empir. Softw. Eng..

[13] Gui J., Mcilroy S., Nagappan M., Halfond W.G. Truth in advertising: The hidden cost of mobile ads for software developers. Proceedings of the 37th International Conference on Software Engineering.

[14] Linares-Vásquez M., Bavota G., Bernal-Cárdenas C., Di Penta M., Oliveto R., Poshyvanyk D. API change and fault proneness: A threat to the success of Android apps. Proceedings of the 2013 9th Joint Meeting on Foundations of Software Engineering.

[15] Sarro F., Al-Subaihin A.A., Harman M., Jia Y., Martin W., Zhang Y. Feature lifecycles as they spread, migrate, remain, and die in app stores. Proceedings of the 2015 IEEE 23rd International Requirements Engineering Conference (RE).

[16] Lim W.C. (1994). Effects of reuse on quality, productivity, and economics. IEEE Softw..

[17] Abdalkareem R., Shihab E., Rilling J. (2017). On code reuse from StackOverflow: An exploratory study on Android apps. Inf. Softw. Technol..

[18] Nasehi S.M., Sillito J., Maurer F., Burns C. What makes a good code example?: A study of programming Q&A in StackOverflow. Proceedings of the 2012 28th IEEE International Conference on Software Maintenance (ICSM).

[19] Kim D., Nam S., Hong J.E. (2019). A dynamic control technique to enhance the flexibility of software artifact reuse in large-scale repository. J. Supercomput..

[20] Wang S., Mao X., Yu Y. (2018). An initial step towards organ transplantation based on GitHub repository. IEEE Access.

[21] Corley C.S., Damevski K., Kraft N.A. (2018). Changeset-based topic modeling of software repositories. IEEE Trans. Softw. Eng..

[22] Chen Z., Xiao L. Agent Component Reuse Based on Semantics Concept Similarity. Proceedings of the 2019 2nd International Conference on Computers in Management and Business.

[23] Ragkhitwetsagul C. Measuring code similarity in large-scaled code Corpora. Proceedings of the 2016 IEEE International Conference on Software Maintenance and Evolution (ICSME).

[24] Diamantopoulos T., Karagiannopoulos G., Symeonidis A. Codecatch: Extracting source code snippets from online sources. Proceedings of the 2018 IEEE/ACM 6th International Workshop on Realizing Artificial Intelligence Synergies in Software Engineering (RAISE).

[25] Freund Y., Iyer R., Schapire R.E., Singer Y. (2003). An efficient boosting algorithm for combining preferences. J. Mach. Learn. Res..

[26] Higgins C., Robles J., Cooper A., Williams S. (2019). Colza: Knowledge-Based, Component-Based Models. Softw. Eng. J..

[27] Patel S., Kaur J. A study of component based software system metrics. Proceedings of the 2016 International Conference on Computing, Communication and Automation (ICCCA).

[28] Jifeng H., Li X., Liu Z. (2005). Component-based software engineering. Component-Based Software Engineering, Proceedings of the International Colloquium on Theoretical Aspects of Computing, Västeras, Sweden, 29 June–1 July 2006.

[29] Linstead E., Bajracharya S., Ngo T., Rigor P., Lopes C., Baldi P. (2009). Sourcerer: Mining and searching internet-scale software repositories. Data Min. Knowl. Discov..

[30] Hoffmann R., Fogarty J., Weld D.S. Assieme: Finding and leveraging implicit references in a web search interface for programmers. Proceedings of the 20th annual ACM symposium on User interface software and technology.

[31] Padhy N., Panigrahi R., Satapathy S.C. (2019). Identifying the Reusable Components from Component-Based System: Proposed Metrics and Model. Information Systems Design and Intelligent Applications.

[32] Bajracharya S., Ossher J., Lopes C. (2014). Sourcerer: An infrastructure for large-scale collection and analysis of open-source code. Sci. Comput. Program..

[33] German D.M., Di Penta M., Gueheneuc Y.G., Antoniol G. Code siblings: Technical and legal implications of copying code between applications. Proceedings of the 2009 6th IEEE International Working Conference on Mining Software Repositories.

[34] Davies J., German D.M., Godfrey M.W., Hindle A. Software bertillonage: Finding the provenance of an entity. Proceedings of the 8th Working Conference on Mining Software Repositories.

[35] Kawamitsu N., Ishio T., Kanda T., Kula R.G., De Roover C., Inoue K. Identifying source code reuse across repositories using lcs-based source code similarity. Proceedings of the 2014 IEEE 14th International Working Conference on Source Code Analysis and Manipulation.

[36] Huang S., Lu Y.Q., Xiao Y., Wang W. Mining application repository to recommend XML configuration snippets. Proceedings of the 2012 34th International Conference on Software Engineering (ICSE).

[37] Diamantopoulos T., Thomopoulos K., Symeonidis A. QualBoa: Reusability-aware recommendations of source code components. Proceedings of the 2016 IEEE/ACM 13th Working Conference on Mining Software Repositories (MSR).

[38] Zhou J., Zhang H. Learning to rank duplicate bug reports. Proceedings of the 21st ACM International Conference on Information and Knowledge Management.

[39] Xuan J., Monperrus M. Learning to combine multiple ranking metrics for fault localization. Proceedings of the 2014 IEEE International Conference on Software Maintenance and Evolution.

[40] Binkley D., Lawrie D. Learning to rank improves IR in SE. Proceedings of the 2014 IEEE International Conference on Software Maintenance and Evolution.

[41] Cox R. (2012). Regular Expression Matching with a Trigram Index or How Google Code Search Worked. https://swtch.com/~rsc/regexp/regexp4.html.

[42] Bruntink M. (2014). An initial quality analysis of the ohloh software evolution data. Electron. Commun. EASST.

[43] McMillan C., Grechanik M., Poshyvanyk D., Fu C., Xie Q. (2011). Exemplar: A source code search engine for finding highly relevant applications. IEEE Trans. Softw. Eng..

[44] Mishne A., Shoham S., Yahav E. Typestate-based semantic code search over partial programs. Proceedings of the ACM International Conference on Object Oriented Programming Systems Languages and Applications.

[45] Keivanloo I., Rilling J., Zou Y. Spotting working code examples. Proceedings of the 36th International Conference on Software Engineering.

[46] Liu T.Y. (2011). Learning to Rank for Information Retrieval.

[47] Kim J., Lee S., Hwang S.w., Kim S. (2010). Towards an intelligent code search engine. Proc. AAAI.

[48] Mcmillan C., Poshyvanyk D., Grechanik M., Xie Q., Fu C. (2013). Portfolio: Searching for relevant functions and their usages in millions of lines of code. ACM Trans. Softw. Eng. Methodol. (TOSEM).

[49] Salton G., Wong A., Yang C.S. (1975). A vector space model for automatic indexing. Commun. ACM.

[50] Manning C.D., Raghavan P., Schütze H. (2008). Scoring, term weighting and the vector space model. Introd. Inf. Retr..

[51] Buse R.P., Weimer W.R. (2009). Learning a metric for code readability. IEEE Trans. Softw. Eng..

[52] Ye X., Bunescu R., Liu C. On the naturalness of software. Proceedings of the IEEE International Conference on Software Engineering.

[53] Wang J., Dang Y., Zhang H., Chen K., Xie T., Zhang D. Mining succinct and high-coverage API usage patterns from source code. Proceedings of the 2013 10th Working Conference on Mining Software Repositories (MSR).

[54] Grahne G., Zhu J. (2003). Efficiently using prefix-trees in mining frequent itemsets. FIMI.

[55] Brin S., Page L. (1998). The anatomy of a large-scale hypertextual web search engine. Comput. Net. ISDN Syst..

[56] Zhong H., Xie T., Zhang L., Pei J., Mei H. (2009). MAPO: Mining and recommending API usage patterns. Proceedings of the European Conference on Object-Oriented Programming.

[57] Holmes R., Walker R.J., Murphy G.C. Strathcona example recommendation tool. Proceedings of the 10th European Software Engineering Conference Held Jointly with 13th ACM SIGSOFT International Symposium on Foundations of Software Engineering.

[58] Jaccard P. (1901). Étude comparative de la distribution florale dans une portion des Alpes et des Jura. Bull. Soc. Vaudoise Sci. Nat..

[59] Niu H., Keivanloo I., Zou Y. (2017). Learning to rank code examples for code search engines. Empir. Softw. Eng..

[60] Gu X., Zhang H., Kim S. Deep code search. Proceedings of the 2018 IEEE/ACM 40th International Conference on Software Engineering (ICSE).

[61] Haldar R., Wu L., Xiong J., Hockenmaier J. (2020). A multi-perspective architecture for semantic code search. arXiv.

[62] Shuai J., Xu L., Liu C., Yan M., Xia X., Lei Y. Improving code search with co-attentive representation learning. Proceedings of the 28th International Conference on Program Comprehension.

[63] Husain H., Wu H.H., Gazit T., Allamanis M., Brockschmidt M. (2019). Codesearchnet challenge: Evaluating the state of semantic code search. arXiv.

